# BCG-Induced DNA Methylation Changes Improve Coronavirus Disease 2019 Vaccine Immunity Without Decreasing the Risk for Severe Acute Respiratory Syndrome Coronavirus 2 Infection

**DOI:** 10.1093/ofid/ofaf007

**Published:** 2025-01-16

**Authors:** Santiago Carrero Longlax, Kent J Koster, Ashish M Kamat, Marisa Lozano, Seth P Lerner, Rebecca Hannigan, Tomoki Nishiguchi, Abhimanyu, Daanish Sheikh, Malik Ladki, Alexandra Portillo, Amrit Koirala, Tajhal D Patel, Zoe Spieler, Aaron B Benjamin, Maxim Lebedev, Theresa U Ofili, Robert W Hutchison, George Udeani, Lynne A Opperman, Gabriel Neal, Anna M Mandalakas, Mihai G Netea, Moshe Arditi, Pablo Avalos, Sandra L Grimm, Cristian Coarfa, Jeffrey D Cirillo, Andrew R DiNardo

**Affiliations:** Global Tuberculosis Program, William T. Shearer Center for Immunobiology, Texas Children's Hospital, Baylor College of Medicine, Houston, Texas, USA; Center for Airborne Pathogen Research and Imaging, Texas A&M School of Medicine, Bryan, Texas, USA; Urology Department, University of Texas MD Anderson Cancer Center, Houston, Texas, USA; Urology Department, University of Texas MD Anderson Cancer Center, Houston, Texas, USA; Scott Department of Urology, Baylor College of Medicine, Houston, Texas, USA; Scott Department of Urology, Baylor College of Medicine, Houston, Texas, USA; Global Tuberculosis Program, William T. Shearer Center for Immunobiology, Texas Children's Hospital, Baylor College of Medicine, Houston, Texas, USA; Global Tuberculosis Program, William T. Shearer Center for Immunobiology, Texas Children's Hospital, Baylor College of Medicine, Houston, Texas, USA; Global Tuberculosis Program, William T. Shearer Center for Immunobiology, Texas Children's Hospital, Baylor College of Medicine, Houston, Texas, USA; Global Tuberculosis Program, William T. Shearer Center for Immunobiology, Texas Children's Hospital, Baylor College of Medicine, Houston, Texas, USA; Global Tuberculosis Program, William T. Shearer Center for Immunobiology, Texas Children's Hospital, Baylor College of Medicine, Houston, Texas, USA; Dan L. Duncan Comprehensive Cancer Center, Baylor College of Medicine, Houston, Texas, USA; Dan L. Duncan Comprehensive Cancer Center, Baylor College of Medicine, Houston, Texas, USA; Global Tuberculosis Program, William T. Shearer Center for Immunobiology, Texas Children's Hospital, Baylor College of Medicine, Houston, Texas, USA; Center for Airborne Pathogen Research and Imaging, Texas A&M School of Medicine, Bryan, Texas, USA; Center for Airborne Pathogen Research and Imaging, Texas A&M School of Medicine, Bryan, Texas, USA; Department of Pharmacy Practice, Texas A&M School of Pharmacy, College Station, Texas, USA; Department of Pharmacy Practice, Texas A&M School of Pharmacy, College Station, Texas, USA; Department of Pharmacy Practice, Texas A&M School of Pharmacy, College Station, Texas, USA; Department of Biomedical Sciences and Center for Craniofacial Research and Diagnosis, Texas A&M School of Dentistry, Dallas, Texas, USA; Primary Care and Rural Medicine, Texas A&M School of Medicine, Bryan, Texas, USA; Global Tuberculosis Program, William T. Shearer Center for Immunobiology, Texas Children's Hospital, Baylor College of Medicine, Houston, Texas, USA; Clinical Infectious Diseases, Research Centre Borstel, Borstel, Germany; Clinical Tuberculosis Unit, German Centre for Infection Research, Borstel, Germany; Department of Internal Medicine and Radboud Center for Infectious Diseases, Radboud University Medical Center, Nijmegen, The Netherlands; Departments of Pediatrics and Biomedical Sciences, Cedars-Sinai Medical Center, Los Angeles, California, USA; Departments of Pediatrics and Biomedical Sciences, Cedars-Sinai Medical Center, Los Angeles, California, USA; Dan L. Duncan Comprehensive Cancer Center, Baylor College of Medicine, Houston, Texas, USA; Department of Molecular and Cellular Biology, Baylor College of Medicine, Houston, Texas, USA; Dan L. Duncan Comprehensive Cancer Center, Baylor College of Medicine, Houston, Texas, USA; Department of Molecular and Cellular Biology, Baylor College of Medicine, Houston, Texas, USA; Center for Airborne Pathogen Research and Imaging, Texas A&M School of Medicine, Bryan, Texas, USA; Global Tuberculosis Program, William T. Shearer Center for Immunobiology, Texas Children's Hospital, Baylor College of Medicine, Houston, Texas, USA; Department of Internal Medicine and Radboud Center for Infectious Diseases, Radboud University Medical Center, Nijmegen, The Netherlands

**Keywords:** BCG vaccine, COVID-19, DNA methylation, innate training, epigenetics

## Abstract

**Background:**

The BCG vaccine induces trained immunity, an epigenetic-mediated increase in innate immune responsiveness. Therefore, this clinical trial evaluated if BCG-induced trained immunity could decrease coronavirus disease 2019 (COVID-19)–related frequency or severity.

**Methods:**

A double-blind, placebo-controlled clinical trial of healthcare workers randomized participants to vaccination with BCG TICE or placebo (saline). Enrollment included 529 healthcare workers randomized to receive BCG or placebo. Primary analysis evaluated COVID-19 disease frequency, while secondary analysis evaluated coronavirus immunity in a subset of participants. Study enrollment ceased early in December 2020 following introduction of COVID-19–specific vaccines.

**Results:**

Study enrollment was halted early, prior to reaching the targeted recruitment, and was not powered to detect a decrease in COVID-19 frequency. Symptomatic COVID-19 occurred in 21 of 263 and 10 of 266 participants in the BCG and placebo arms, respectively (*P* = .50, Fisher exact test). Participants vaccinated with BCG, but uninfected with severe acute respiratory syndrome coronavirus 2 (SARS-CoV-2), demonstrated increased coronavirus vaccine immunity (increase spike-inducible levels of tumor necrosis factor, interleukin 6, and interleukin 1β) 12 months after BCG vaccination compared to participants receiving placebo. Immune responsiveness to SARS-CoV-2 antigens correlated with BCG-induced DNA methylation changes.

**Conclusions:**

Due to early study closure, the study was not powered to evaluate COVID-19 frequency. Secondary analysis demonstrated that 12 months following vaccination, BCG increased coronavirus vaccine immunity compared to those who did not receive BCG. This increase in COVID-19 vaccine immunity correlated with BCG-induced DNA methylation changes.

## Teaser

BCG-induced DNA methylation enhances COVID-19 vaccine immunity without lowering SARS-CoV-2 infection risk. This study suggests that BCG vaccination induces epigenetic changes that improve immune responses to subsequent COVID-19 vaccination.

In early 2020, at the start of the coronavirus disease 2019 (COVID-19) pandemic, COVID-19–specific vaccines were far on the horizon, and it was assumed that such vaccines would not be imminently available. Historically, vaccine development took years. The fastest vaccine previously developed was the mumps vaccine created by Dr. Maurice Hilleman from a strain of mumps collected from his daughter Jeryl Lynn, with vaccine development spanning 4 years from 1963 to 1967 [1]. Therefore, while awaiting the development of a COVID-19–specific vaccine, researchers questioned if induction of trained immunity might provide decreased disease frequency or severity of COVID-19.

Trained immunity, the antigen-nonspecific immune response to heterologous immune stimulation, was first described by George Mackaness from 1962 to 1964 [1, 2]. Mackaness’ experiments found that macrophages exposed to one stimulation by one microbial stimulus (often bacillus Calmette-Guérin [BCG]) would display an increased immune response to another, unrelated, microbial challenge. Over the past decade, studies have defined the epigenetic mechanism for innate training. After exposure to an unrelated antigenic stimulation, frequently either BCG or β-glucan, epigenetic changes to chromatin conformation result in increased immune response to unrelated future secondary challenges. For example, BCG induces H3K4 trimethylation at the *TNF* and *IL6* promoters, resulting in macrophages that produce more tumor necrosis factor (TNF) and interleukin 6 (IL-6) protein when they are stimulated with *Candida* or *Staphylococcus* months later [3]. In neonates, BCG induces DNA methylation changes in innate immunity and interferon genes that last at least 14 months postvaccination [4].

Historically, Mackaness’ work on “acquired cellular resistance” and studies over the last decade on trained immunity have evaluated how exposure to one bacterial species increased responsiveness to unrelated bacterial species. In addition, BCG also improves antiviral immunity. For example, BCG given before the yellow fever vaccine virus decreases the time an individual is viremic [5]. Similarly, study participants who receive BCG before an influenza vaccination display increased hemagglutinin A titers and interferon gamma (IFN-γ) production [6]. Therefore, it was postulated that vaccination with BCG might be capable of decreasing COVID-19 severity or disease susceptibility.

We therefore implemented a randomized, blinded clinical trial of healthcare workers (HCWs) and high-risk individuals to evaluate if BCG vaccination could decrease COVID-19 frequency or severity. Based on existing evidence demonstrating a decrease in viral upper respiratory tract infections after BCG, a priori power analysis assuming a 60% BCG vaccine efficacy indicated that the study would require 1800 participants to be followed for 12 months [7]. When the COVID-19–specific vaccine was released in December 2020, this study was halted early, and the following presents epigenetic and immune secondary outcomes.

## MATERIALS AND METHODS

### Study Population and Trial Design

**Figure 1.**
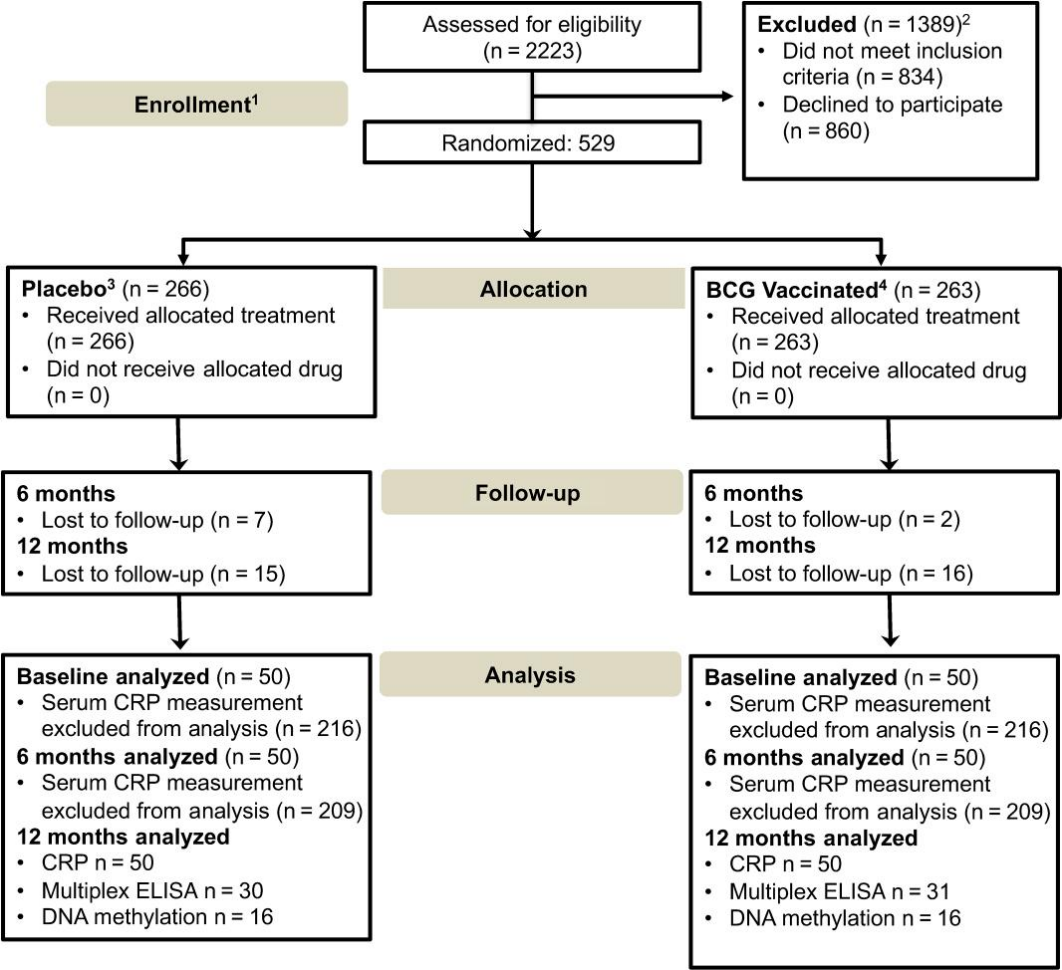
Consolidated Standards of Reporting Trials (CONSORT) diagram of the clinical study design and analyses. ^1^Of 2023 healthcare workers who applied online, 1300 were eligible, and 529 were enrolled. Participants included those working in hospitals or medical centers/clinics, as well as first responders (paramedics, firefighters, law enforcement). ^2^People with human immunodeficiency virus, known immunodeficiency, neutropenia, or participants on immunotherapy, oral or intravenous steroids, or immunosuppressants. ^3^Placebo: 0.9% sodium chloride solution, 0.1 mL, 1-time administration intradermally in the deltoid area. ^4^BCG-vaccinated group: TICE BCG live strain (Merck), US Food and Drug Administration approved, 0.1 mL (∼2 × 10^5^ colony-forming units), 1-time administration intradermally in the deltoid area. ^5^Participants were followed up to assess for symptoms and adverse events. Optional blood donations were collected for the isolation of peripheral blood mononuclear cells and for serological analysis of severe acute respiratory syndrome coronavirus 2 antibodies using dried blood spot samples. Additionally, participants’ messenger RNA vaccine history was documented. Abbreviations: CRP, C-reactive protein; ELISA, enzyme-linked immunosorbent assay.

The protocol for the BCG as Defense Against SARS–COVID-19 Clinical Trial has been published [7], and the study was conducted at 5 sites in the United States (Texas A&M University, College Station, Texas; Texas A&M School of Dentistry, Dallas, Texas; Baylor College of Medicine, Houston, Texas; MD Anderson Cancer Center, Houston, Texas; and Cedars-Sinai Medical Center, Los Angeles, California). The study was a randomized, double-blind, placebo-controlled trial in adults considered to be at high risk, specifically HCWs and other personnel with high occupational exposure to COVID-19 or individuals with comorbidities detailed in the previously published protocol. The study was registered at ClinicalTrials.gov as NTC04348370 after approval from local and central institutional review boards. Informed electronic, written consent was obtained from all participants and saved with participants’ information in Health Insurance Portability and Accountability Act–compliant Research Electronic Data Capture (REDCap) software. Participants were eligible if they were adults ≥18 years old and at high risk for severe acute respiratory syndrome coronavirus 2 (SARS-CoV-2) exposure (Figure 1). Participants were ineligible if there was a known allergy to the BCG vaccine or components, active *Mycobacterium tuberculosis* or other mycobacterial infection, fever within the last 24 hours, aged >75 years, pregnant or planning pregnancy within 30 days of study enrollment, breastfeeding, immunocompromised, or with active bacterial or viral infections. Participants were also excluded if they had previously documented COVID-19, active solid malignancy, lymphoma, or other cancer. Potential participants were screened using an online REDCap questionnaire with a study member confirming eligibility criteria in person or over the phone. The REDCap system randomized participants to receive either the BCG or placebo (saline) administered subcutaneously in the deltoid. Participants received the placebo or BCG (0.1 mL TICE, Merk) vaccine after an optional 12 mL of blood donation. Participants were followed for 12 months, completing a weekly survey using the REDCap app to document SARS-CoV-2 infection and symptoms. The first participants received intervention on 6 June 2020. The a priori power analysis assumed a 60% vaccine efficacy, requiring 1800 participants. A data and safety monitoring board (DSMB) regularly met to review enrollment, adverse events, and outcomes. Adverse events were graded as previously described [7] using the US Food and Drug Administration and World Health Organization guidance on vaccine-related adverse events. New participant enrollment was halted in December 2020 when a COVID-19–specific vaccine was developed. We calculated the interquartile range (IQR) to assess the variability in the incidence and severity of COVID-19 in both the BCG and placebo groups during the follow-up period. Given the limited sample size, we employed Fisher exact test to evaluate the association between treatment groups and outcomes.

### Blood Sample Collection and Analysis

Blood samples were obtained at baseline (before treatment administration) and 6 and 12 months postintervention (Figure 1). Additionally, samples were obtained if, during the survey screenings, participants provided responses indicating a potential SARS-CoV-2 infection. Throughout the study, all research personnel, except for the established DSMB members, remained blinded to participant group assignments. The DSMB had access to unblinded data to monitor safety and efficacy. Research personnel were unblinded only after analysis was completed, at which point they identified which groups received the BCG or placebo intervention. Due to pandemic concerns, the institutional review board required blood donations to be voluntary to minimize constraints on HCWs. After blood collection, peripheral blood mononuclear cells (PBMCs) were isolated and thereafter stored in liquid nitrogen. Dry-blood samples and plasma were preserved in freezers for further analysis (Supplementary Figure 1). Asymptomatic disease was identified in subjects by the presence of antibodies by enzyme-linked immunosorbent assay (ELISA) against SARS-CoV-2 spike proteins S1/S2 and nucleocapsid (Thermo Fisher Scientific, Waltham, Massachusetts), similar to that previously described [8].

C-reactive protein (CRP) levels were measured in a randomly selected subset of participants comprising 50 individuals from each study arm (BCG or placebo) at baseline, 6 months, and 12 months, using the Human C-Reactive Protein/CRP Duoset ELISA (R&D Systems Biotechne), as shown in Supplementary Figure 1.

Multiplex ELISA was performed as previously described [9, 10]. In brief, PBMCs from 72 participants were randomly selected and thawed with a minimum viability of 70% required for use. The samples included an equal number of male and female participants and was pair-matched with both baseline and 12-month samples. In total, 144 vials were thawed and used. After confirming viability, 1 × 10^6^ PBMCs were aliquoted into 4 separate wells and then stimulated with (1) nil (R10 media control consisting of RPMI and 10% pooled human AB serum); (2) lipopolysaccharide (LPS; 10 ng/mL; R&D Systems); (3) overlapping peptide pools of the SARS-CoV-2–specific spike protein (1 μg/mL; Peptivator COVID S, Miltenyi Biotec); and (4) overlapping peptide pools of the coronavirus nucleocapsid (1 μg/mL, Peptivator COVID N, Miltenyi Biotec) that is nonspecific to SARS-CoV-2 and overlaps with many other coronavirus strains. After overnight stimulation, the supernatant was removed, and multiplex ELISA was performed using the Human Inflammation Panel 1 (LegendPlex assay, BioLegend). As part of quality control, samples that did not have a minimum of a 1.5-fold increase in TNF-α production in response to LPS stimulation compared to the nil control were removed from downstream analysis. Five participants from the BCG group and 6 from the placebo group were excluded due to the absence of a detectable TNF-α response following LPS stimulation. The fold change at 12 months over placebo was calculated for each cytokine. The Mann-Whitney *U* test, which does not assume normality, was then used to compare the placebo and the BCG-vaccinated groups for each cytokine analyzed. To control for multiple comparisons, the Holm-Sidak correction was applied. A Fisher exact test was used to evaluate COVID-19 rates between the 2 study groups.

### DNA Methylation

DNA methylation was performed as previously described [11, 12]. A priori sample size power analysis, and our previous publications, demonstrated that n = 16 per group would allow discriminating DNA methylation differences [10–13]. Each group consisted of 16 samples, with 8 females and 8 males. In the placebo group, the age distribution showed a Q1 (25th percentile) of 55.65 years and a Q3 (75th percentile) of 66.64 years. Similarly, in the BCG group, the age distribution was the same, with a Q1 of 55.65 years and a Q3 of 66.64 years. For body mass index (BMI), the BCG group had a Q1 of 32.92 kg/m^2^ and a Q3 of 62.59 kg/m^2^, while the placebo group had a BMI Q1 of 33.88 kg/m^2^ and a Q3 of 61.18 kg/m^2^. In brief, after thawing PBMCs and confirming >70% viability, DNA was isolated using the DNAeasy kit (Qiagen). DNA quality and quantity were evaluated using the Agilent TapeMeasure. DNA methylation was then determined using the Illumina Infinium MethylEPIC array and processed as previously described. Pathway analysis was implemented using the Gene Ontology compendium and gene set enrichment analysis via the Molecular Signatures Database (MSigDB) approach using hypergeometric distribution; the Benjamini-Hochberg multiple hypothesis testing correction was used. For each gene, DNA methylation was aggregated using the median CpG DNA methylation value at transcription start site (TSS) based on the MethylEpic array annotation. Next, we computed a methylation score based on gene sets as follows: first, methylation at 1 gene TSS was z-score transformed across all the samples; next, the z-scores were added for each gene in the gene set, and we termed this result a gene set summed z-score. We computed summed z-scores for each pathway in the Hallmark compendium; the correlation between pathway summed z-scores and clinical traits was determined using the Pearson correlation coefficient, with significance achieved at *P* < .05.

## RESULTS

From Los Angeles (California) and College Station, Austin, Round Rock, Dallas, Fort Worth, and Houston (Texas), 2223 HCWs were invited and screened, with 529 participants enrolled from June 2020 to December 2020, of whom 266 participants received saline placebo and 263 received BCG vaccine. The demographics, age, and comorbidity status of the participants are presented in Table 1.

**Table 1.** Baseline and 12-Month Follow-up Epidemiological Characteristics of Enrolled Participants

Characteristic	Placebo	BCG Group	Total
	(n = 266)	(n = 263)	(N = 529)
Age, y, mean (SD)	46.60 (12.4)	47.01 (12.30)	46.81 (12.37)
Sex			
Female	181 (68)	167 (63.5)	348 (65.8)
Male	85 (32)	96 (36.5)	181 (34.2)
BMI, kg/m^2^, median (range)	26.2 (14.52–54.9)	25.83 (13.9–55.29)	26.1 (13.9–55.29)
Race			
Black or African American	8 (3)	4 (1.5)	12 (2.3)
White	213 (80)	212 (80.6)	425 (80.3)
Native Hawaiian or other Pacific Islander	1 (0.4)	2 (0.8)	3 (0.6)
Asian	27 (10.2)	31 (11.7)	58 (11)
Native American/ American Indian	1 (0.4)	2 (0.8)	3 (0.6)
More than 1 race	16 (6.0)	12 (4.6)	28 (5.3)
Ethnicity			
Hispanic or Latino	39 (14.7)	23 (8.7)	62 (11.7)
Not Hispanic or Latino	227 (85.3)	240 (91.3)	467 (88.3)
Prior history of BCG vaccination			
No history	216 (81.2)	204 (77.6)	420 (79.4)
Previous vaccination	35 (13.2)	28 (10.6)	63 (11.9)
Unknown	15 (5.6)	31 (11.8)	46 (8.7)
Comorbid conditions			
Asthma (current)	36 (13.5)	35 (13.3)	71 (15.8)
Diabetes (ever diagnosed)	11 (4.1)	17 (6.5)	28 (5.3)
Hypertension (ever diagnosed)	53 (19.9)	56 (21.3)	109 (20.6)
Hypercholesterolemia	54 (20.3)	72 (27.4)	126 (23.8)
Heart disease (ever diagnosed)	4 (1.5)	4 (1.5)	8 (1.5)
Other	45 (16.9)	41 (15.6)	86 (16.3)
Smoking (>3 cigarettes per wk)	43 (16.2)	38 (14.4)	81 (15.3)

Data are presented as No. (%) unless otherwise indicated. Table shows coronavirus disease 2019 severity in participants diagnosed with severe acute respiratory syndrome coronavirus 2 (SARS-CoV-2) infection by antigen or reverse-transcription polymerase chain reaction. Participants were classified as having mild, moderate, severe, or critical illness according to the 2022 SARS-CoV-2 National Institutes of Health guidelines.

Abbreviations: BMI, body mass index; IQR, interquartile range; SD, standard deviation.

The intervention was well tolerated (Supplementary Table 1) with no grade 3 or 4 serious adverse events. Study enrollment was halted before full enrollment due to the earlier than expected release of the COVID-19–specific vaccines in December 2020. Enrolled participants were followed as per study protocol; in the placebo group, 15 (5.6%) participants dropped out and declined to continue. Similarly, in the BCG group, 16 (6.0%) participants dropped out and declined to continue (Figure 1). Before 19 December 2020, when the COVID-19–specific vaccine became available, 21 and 17 participants developed symptomatic COVID-19 in the BCG and placebo arms, respectively (Table 2; *P* = .50, Fisher exact test). During the 12-month follow-up, including the period before and after the COVID-19–specific vaccine, no study participants had severe COVID-19 or were hospitalized for COVID-19, and 8 participants had asymptomatic disease detected by screening after exposure (Table 2). Among all participants, only 38 participants developed symptomatic COVID-19 disease; of these, 8 (1.5%) participants had moderate disease, and 30 (5.7%) participants developed mild COVID-19 disease. In summary, there was no statistical difference in COVID-19 disease frequency among participants who received BCG compared to placebo.

**Table 2.** Clinical Outcomes Between Baseline and 12 Months

Outcome	Placebo	BCG Group	Total
	(n = 266)	(n = 263)	(N = 529)
SARS-CoV-2 (PCR^+^)	18 (6.8% [3.8%–9.8%])	24 (9.1% [5.6%–12.6%])	42 (7.9%)
SARS-CoV-2 (antigen)	3 (1.13% [0%–2.40%])	1 (0.38% [0%–1.12%])	4 (0.75%)
COVID-19 severity			
Asymptomatic	4 (1.5% [.04%–2.97%])	4 (1.5% [.04%–3.0%])	8 (1.5%)
Mild illness	12 (4.5% [2.02%–7.01%])	18 (6.8% [3.79%–9.90%])	30 (5.7%)
Moderate illness	5 (1.9% [.25%–3.51%])	3 (1.1% [0%–2.42%])	8 (1.5%)
Severe illness	0 (0%)	0 (0%)	0 (0%)
Critical illness	0 (0%)	0 (0%)	0 (0%)
Hospitalization	0 (0%)	0 (0%)	0 (0%)

Data are presented as No. (% [95% confidence interval]) for COVID-19 outcomes in the placebo and BCG groups. Participants were classified as having mild, moderate, severe, or critical illness based on the 2022 SARS-CoV-2 National Institutes of Health guidelines.

Abbreviations: COVID-19, coronavirus disease 2019; PCR^+^, polymerase chain reaction positive; SARS-CoV-2, severe acute respiratory syndrome coronavirus 2.

Virtually all participants (99%) documented receiving a COVID-19–specific messenger RNA vaccine, a median of 132 days (IQR, 27–267 days) after receiving BCG or placebo. Previous publications demonstrated that BCG given before another vaccine increases immunity to the other vaccine [14, 15]. Historically, vaccine adjuvants (for example, Freund adjuvant) used mycobacterial components to boost vaccine immunity. Studies have demonstrated that COVID-19 vaccine immunity wanes with time [16]. Immune analysis therefore evaluated if individuals who did not develop symptomatic COVID-19 and had negative SARS-CoV-2 antibody serology had long-term vaccine immunity that differed between the BCG and placebo groups.

**Figure 2.**
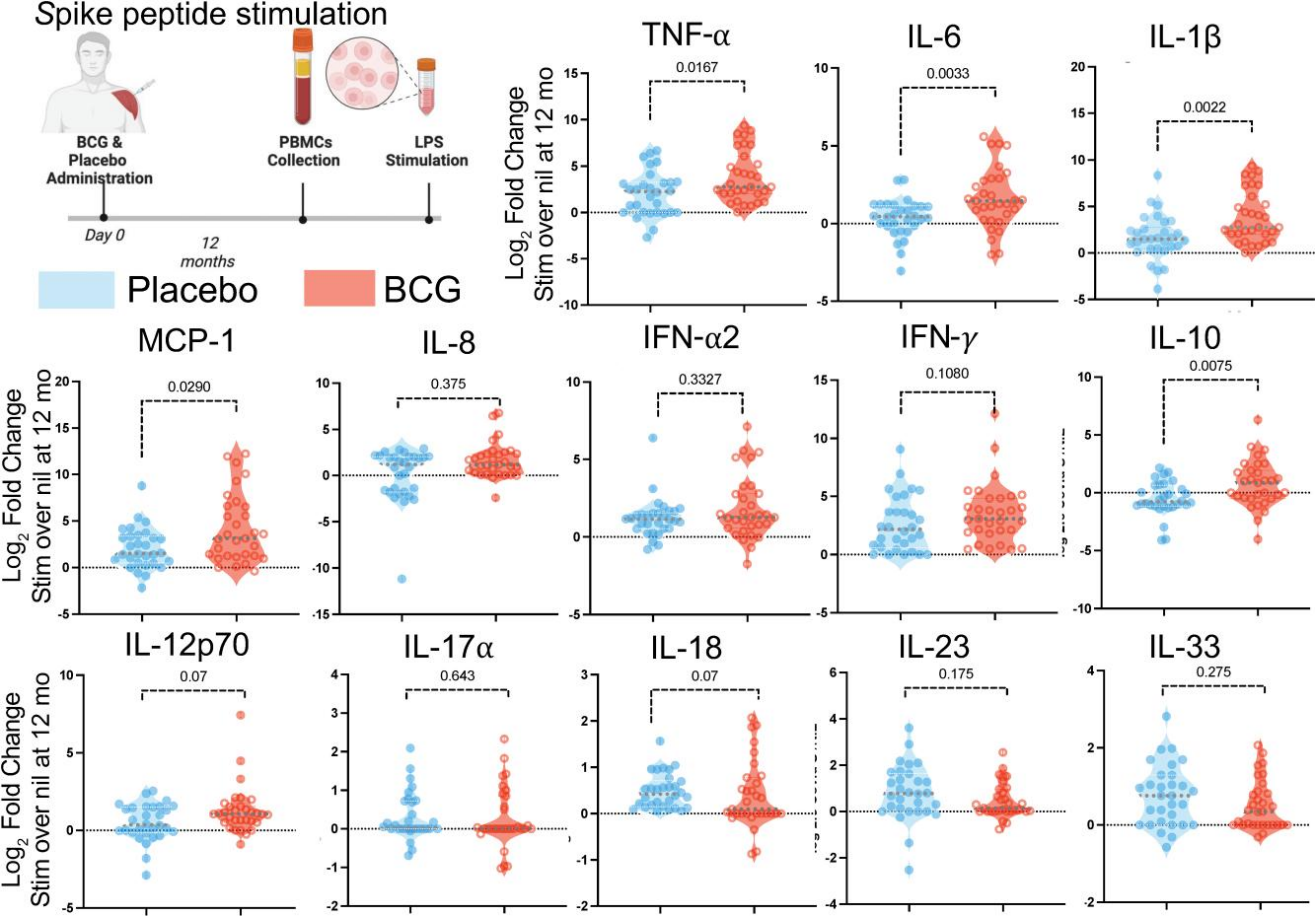
BCG augments severe acute respiratory syndrome coronavirus 2 (SARS-CoV-2) spike immunity. Peripheral blood mononuclear cells (PBMCs) were collected 12 months after BCG vaccination (n = 31; red) or placebo vaccination (n = 30; light blue). The cells were frozen immediately after collection and later thawed for batch immune evaluation. PBMCs were left unstimulated or stimulated overnight with overlapping peptide pools of the spike protein found specifically in SARS-CoV-2 with the supernatant evaluated by multiplex enzyme-linked immunosorbent assay (LegendPlex, BioLegend). Responses are expressed as log_2_ fold change of stimulated over unstimulated with the center line representing the median. Estimation effect measured by Mann-Whitney test comparing cytokine response in both groups with Holm-Sidak correction. Abbreviations: IFN, interferon; IL, interleukin; LPS, lipopolysaccharide; MCP-1, Monocyte Chemotactic protein 1; PBMCs, peripheral blood mononuclear cells; TNF, tumor necrosis factor.

Participants completed 12 months of follow-up and provided self-administered finger-stick dried blood spots monthly, and had the option to donate 12 mL of blood at baseline, 6 months, and 12 months. Supplementary Figure 1 demonstrates the experimental timeline and layout. Trained immunity has previously been demonstrated to last up to 12 months in some studies [17]. In this evaluation of bulk PBMCs stimulated with LPS 12 months after treatment administration, immune cells from participants who had previously received BCG vaccine demonstrated a significant increase in interleukin 8 (IL-8) production (median LPS-induced IL-8 of 3.2 in the BCG arm vs 2.4 in the placebo arm; *P* = .02, Mann-Whitney test). In contrast, there was no statistical increase in IL-6, TNF-α, or other cytokines or chemokines previously associated with trained immunity (Supplementary Figure 2; IL-8 two-fold change at 12 months, *P* < .05, Mann-Whitney test). Furthermore PBMCs from BCG-vaccinated participants upon SARS-CoV-2–specific spike stimulation demonstrated increased production of IL-6, interleukin 1β (IL-1β), TNF-α, interleukin 10 (IL-10), and monocyte Chemotactic protein 1 (MCP-1) compared to the placebo group 12 months after receiving the BCG vaccine but not for IFN-γ, IL-8, or interferon alpha (IFN-α) (Figure 2; *P* > .05, respectively, Mann-Whitney test).

**Figure 3.**
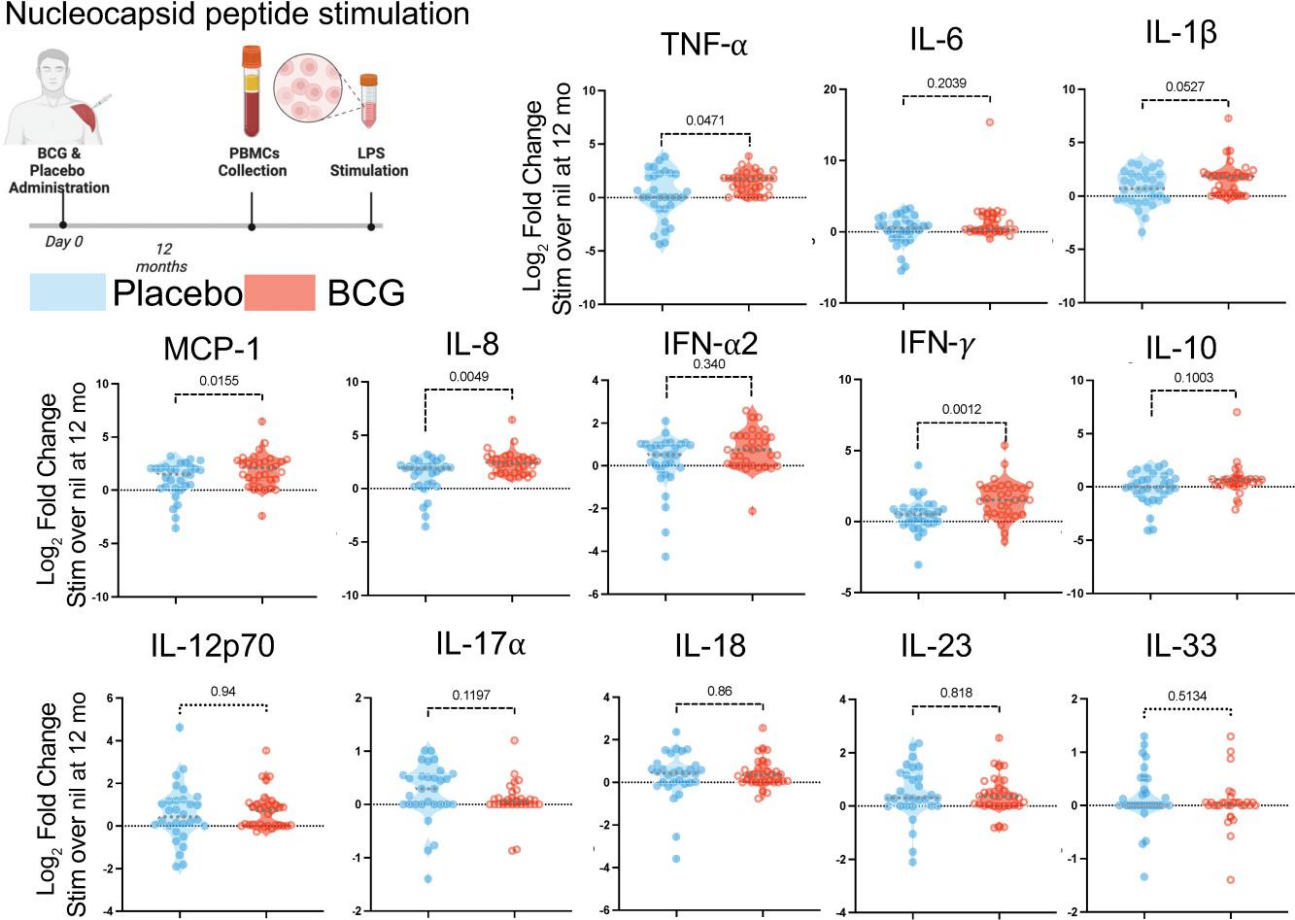
BCG augments coronavirus nucleocapsid immunity. Peripheral blood mononuclear cells (PBMCs) were collected 12 months after BCG (n = 31) or placebo (n = 30) vaccination, frozen, and later thawed for batch immune evaluation. PBMCs were left unstimulated or stimulated overnight with overlapping peptide pools of the nucleocapsid found in severe acute respiratory syndrome coronavirus 2 and other coronaviruses with the supernatant evaluated by multiplex enzyme-linked immunosorbent assay (LegendPlex, BioLegend). Responses are expressed as log_2_ fold change of stimulated over unstimulated with the center line representing the median. Estimation effect measured by Mann-Whitney test comparing cytokine response in both groups with Holm-Sidak correction. Abbreviations: IFN, interferon; IL, interleukin; LPS, lipopolysaccharide; MCP-1, Monocyte Chemotactic protein 1; PBMCs, peripheral blood mononuclear cells; TNF, tumor necrosis factor.

To evaluate generic, non-COVID-19–specific coronavirus immunity 12 months after receiving BCG vaccine or placebo, PBMCs were also stimulated with overlapping peptide pools of the nucleocapsid protein that is shared between most coronaviruses. In comparison to the placebo arm, BCG-vaccinated participants demonstrated increased IFN-γ, TNF-α, IL-8, and MCP-1 production in response to nucleocapsid peptides (Figure 3; *P* < .05, Mann-Whitney test). Previous studies had demonstrated that in addition to augmenting antigen-induced immune responsiveness, BCG vaccination decreased basal inflammation [18, 19]. In this cohort, CRP was statistically similar at baseline (median, 1.5 vs 2.7; *P* = .47, Mann-Whitney test) and at 6 months (median, 1.9 vs 4.8; *P* = .0526) and 12 months after BCG vaccination (median, 1.52 vs 2.0; *P* = .24; Supplementary Figure 3).

**Figure 4.**
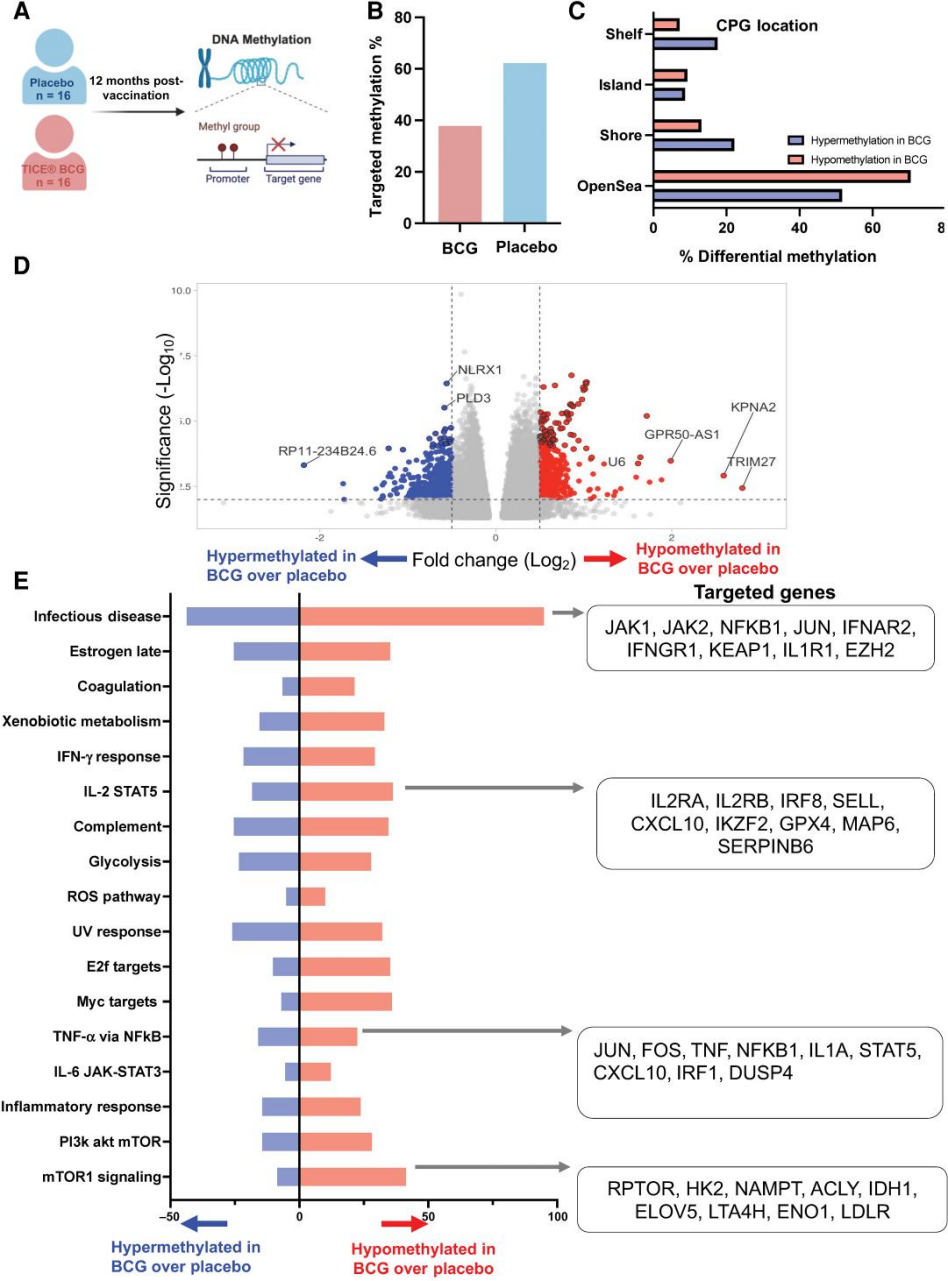
BCG-induced DNA methylation changes. *A*, 12 months after BCG or placebo vaccination, 32 participants donated additional blood for DNA methylation. *B*, Location of BCG-induced hyper- and hypomethylation. *C*, Volcano plot demonstrating select hyper- and hypomethylated genes 12 months after BCG vaccination (a complete list of differential methylated genes is shown in Supplementary Table 2). *D*, Overrepresentation analysis depicting the enriched hyper- and hypomethylated hallmark gene pathways. *E*, Select pathways and genes hypomethylated 12 months after BCG vaccination (a complete list of all enriched pathways is shown in Supplementary Table 2). Abbreviations: CpG, The majority of DNA methylation occurs on cytosines that precede a guanine nucleotide; IFN, interferon; IL, interleukin; mTOR, mammalian target of rapamycin; ROS, reactive oxygen species; TNF, tumor necrosis factor; UV, ultraviolet.

**Figure 5.**
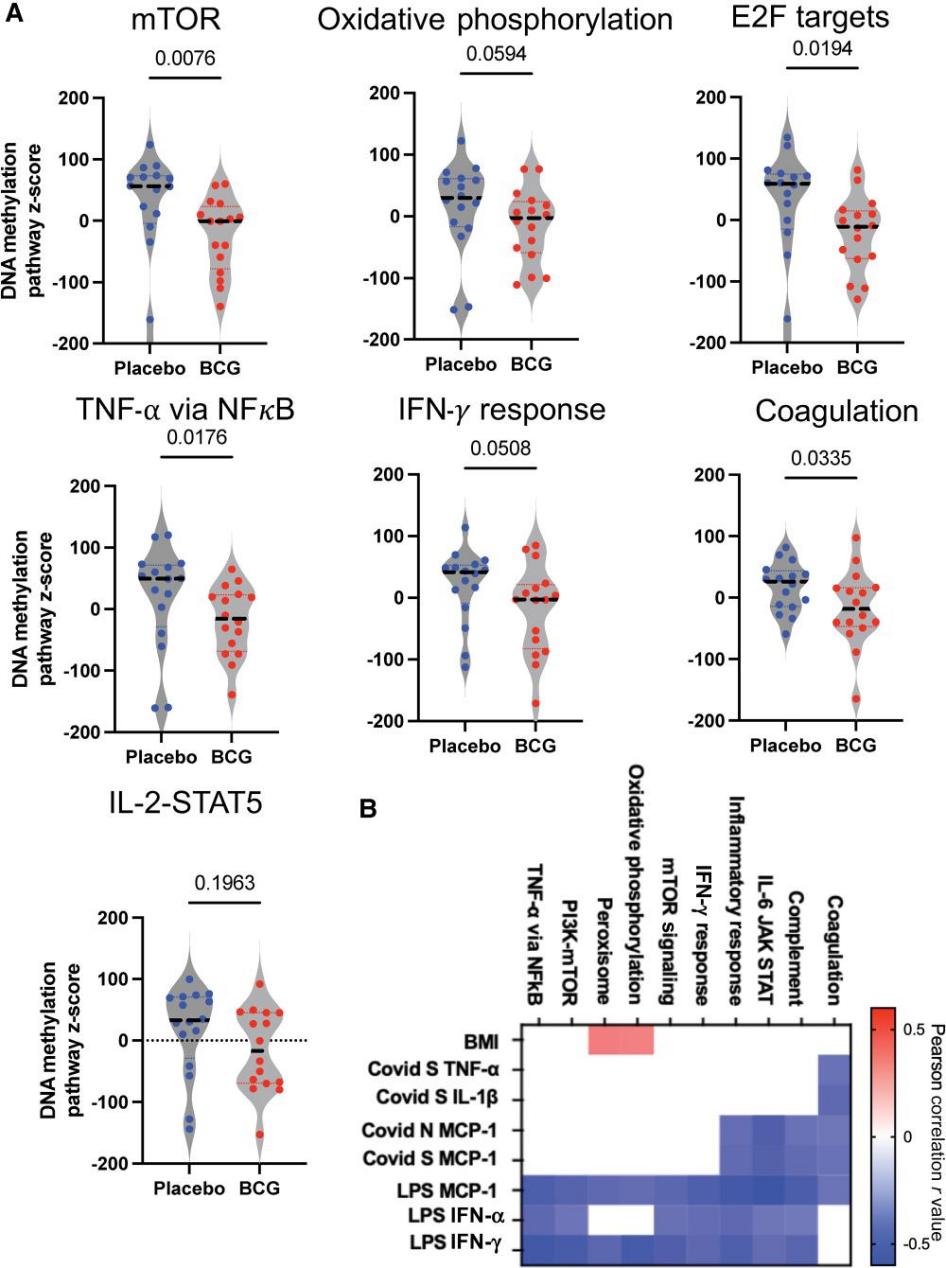
BCG-induced DNA methylation changes correlate with coronavirus disease 2019 and lipopolysaccharide-induced immunity. *A*, The DNA methylation summed z-score for each pathway, with center line representing the median z-score: estimation effect measured by 2-way analysis of variance comparing placebo vs BCG. *B*, Heatmap showing Pearson correlations between the DNA methylation summed z-scores for selected pathways (columns) and host immunity markers (rows) induced by lipopolysaccharide, spike, or nucleocapsid peptides. Only significant correlations are displayed (*P* < .05). Red indicates positive correlations, blue indicates negative correlations, and white represents nonsignificant correlations. Abbreviations: BMI, body mass index; IFN, interferon; IL, interleukin; LPS, lipopolysaccharide; MCP-1, Monocyte chemtotactic protein 1; mTOR, mammalian target of rapamycin; N, nucleocapsid; S, spike; TNF, tumor necrosis factor.

Remodeling of the epigenetic landscape is one mechanism that underlies the antigenic nonspecific trained immunity induced by BCG [3, 6]. Therefore, to evaluate the long-lasting epigenetic changes induced by BCG 12 months after vaccination, DNA methylation status was evaluated using the DNA Methyl EPIC array (Illumina) on 16 age- and sex-matched participants per group (Figure 4*A*). Compared to participants who received placebo, BCG-vaccinated participants demonstrated 5500 hypermethylated genes and 3331 hypomethylated genes using a cut-off of genes with >2 log_2_ fold change and *P* < .05 (Figure 4*B* and 4*C*; all differentially methylated probes are listed in Supplementary Table 2). BCG predominantly induced a demethylation in genes related to mTOR, Myc, E2F, IL-2–STAT5, and infectious disease pathways (Figure 4*D* and 4*E*). To quantify DNA methylation at the pathway level, the summed z-scores of m-values were evaluated, identifying a decrease in DNA methylation in mTOR, E2F, TNF via NFκB, and IFN-γ, and coagulation pathways (Figure 5*A*; *P* < .05, analysis of variance). The pathway-level DNA methylation scores were compared to the antigen-induced log_2_ fold change, identifying an inverse correlation between DNA methylation and antigen-induced immunity. Specifically, increasing BMI correlated with increased DNA methylation in the oxidative phosphorylation and peroxisome pathways, while the coronavirus nucleocapsid and spike-induced immunity inversely correlated with DNA methylation for coagulation, complement, IL-6–JAK–STAT, and the inflammatory pathways (Pearson correlation *r* = −0.37 to −0.47, *P* < .05). Similarly, LPS-induced production of MCP-1, IFN-α, and IFN-γ inversely correlated with DNA methylation of the TNF-NFκB, mTOR, IFN-γ, inflammatory, and IL-6–JAK–STAT pathways (Pearson correlation *r* = −0.33 to −0.59, *P* < .05; Figure 5*B*).

## DISCUSSION

Vaccination of American HCWs with BCG did not decrease the frequency of COVID-19 disease. No conclusions can be drawn regarding the effects of BCG on disease severity, as most participants had mild disease and no participants had severe disease. However, BCG vaccination induced epigenetic (DNA methylation) changes that inversely correlated with increased COVID-19 vaccine immunity. In 1962, George Mackaness described the antigen-nonspecific nature of BCG, describing it as “acquired cellular resistance” [2]. In the 1940s, Jules Freund recognized the immunogenic potential of heat-killed mycobacteria as a vaccine adjuvant [15, 20]. More recently, trained immunity has been defined as the antigen-agnostic long-term adaptation of innate immune cells induced after infections or vaccinations to mount a faster and stronger immune response to an unrelated infection [21]. Mechanistic studies have identified long-lasting changes in cellular metabolism and epigenetic landscape to underlie trained immunity, and studies have demonstrated that humans who received BCG developed trained immunity that improved antiviral immunity to influenza and yellow fever [5, 22, 23]. This study demonstrates that BCG given months before a COVID-19–specific vaccine modestly increases coronavirus immunity in a manner that correlates with DNA hypomethylation.

Similar to other clinical trials [24], this study demonstrated no decrease in COVID-19 disease frequency among the HCWs who received BCG. There are likely several reasons for this. First, due to the unexpectedly rapid release of COVID-19–specific vaccines, the study did not achieve the targeted enrollment needed to evaluate BCG's effect on COVID-19 incidence. In a study of almost 4000 HCWs in Brazil, Europe, and Australia, BCG offered no protection against symptomatic COVID-19 [24]. Similarly, several large studies in elderly individuals vaccinated with BCG failed to demonstrate protection against COVID-19. Differences in the immune status of the individuals at the time of vaccination, different BCG strains, or differences in the study populations may explain some variation. Further, studies have demonstrated heterogeneity in individual response to BCG vaccination, for example with time of day and sex partially predicting BCG-induced trained immunity [18, 25].

Like previous studies, these results suggest an intimate relationship between cellular metabolism, epigenetic changes, and immune function. Studies dating back to the 1970s had demonstrated that BCG-induced training requires cellular oxidation [26]. Modern studies have demonstrated that the AKT-mTOR pathway and increased glycolysis is required for BCG-induced trained immunity [25]. Similarly, these studies demonstrate BCG-induced DNA methylation in metabolism-related genes (*RPTOR*, *HK2*, *NAMPT*, *ACLY*, *IDH*, *ELOV5*, *NLRX1*) and pathways (mTOR, PI3K, and Myc). The causal relationship between cellular metabolism and epigenetic changes still needs to be clarified, but initial work demonstrates a bidirectional relationship. Specifically, cancer and immunology studies have demonstrated that shifts in tricarboxylic acid (TCA) cycle metabolites (succinate, 2-HD, and -ketoglutarate) or NAD^+^:NADH ratios direct epigenetic changes via -ketoglutarate-dependent dioxygenases, sirtuins, and the NuRD (nucleosome remodeling and deacetylase) complex [27–29]. However, BCG also induces epigenetic changes that regulate cellular metabolism [30, 31]. There is evidence suggesting that the differential methylation of genes related to innate immune pathways, such as NLRX1, supports the relationship between the BCG vaccine's effect on oxidative phosphorylation and pathogen recognition through danger associated molecular patterns/pathogen associated molecular patterns (PAMPs). In vitro studies have shown that NLRX1, a mitochondrial PAMP, increases during viral infections, such as human immunodeficiency virus type 1 (HIV-1), resulting in increased oxidative phosphorylation and glycolysis. Additional evidence suggests that inhibiting mitochondrial complexes and suppressing oxidative phosphorylation could result in less viral replication and higher CD4 T-cell recovery in HIV [30]. Future studies should further investigate how BCG-induced hypermethylation of NLRX1 regulates immune responsiveness and future vaccine immunity.

Recognized limitations of the study include the low frequency of disease and the absence of severe disease. Adherence to masking and social distancing among the HCW participants likely contributed to the low total incidence of disease, which limited analytic power. The absence of severe disease further limits the ability to assess the impact of BCG vaccination on the severity of COVID-19. None of the published studies to date were powered to reach a conclusion regarding BCG effect on COVID-19 disease severity. Several randomized trials assessing the engineered BCG strain VMP-1002 and the measles-mumps-rubella vaccine [28, 29] support the proposition that induction of trained immunity may protect against disease severity, but not the total number of infections. Additional limitations include the corollary nature of the study. Studies using severe combined immunodeficiency mice lacking adaptive immunity have demonstrated a lymphocyte-independent nature of trained immunity [32]; however, recent studies have clarified the complexity of the subject, demonstrating a cross-talk between adaptive and innate immunity [32–34]. Harnessing the safe induction of trained immunity to augment disease-specific vaccine should improve the duration and efficacy of vaccines. Other studies have demonstrated that only approximately 50% of healthy individuals develop trained immunity, with sex, preexisting chromatin architecture, time of vaccine administration, and seasonality predicting BCG-induced training in healthy individuals [19, 35]. Understanding why certain individuals are and are not trainable is needed to help improve rational vaccine design.

In summary, epigenetic and immune analysis completed in this study confirm that BCG induces long-lasting DNA methylation changes and that these DNA methylation changes correlate with both nonspecific coronavirus (nucleocapsid-induced) and SARS-CoV-2–specific (spike-induced) immunity. Considering the waning effect of COVID-19–specific vaccination, future studies could evaluate if trained immunity can amplify and therefore help prolong vaccine immunity. Similarly, a better understanding of the heterogeneity of BCG-induced trained immunity could potentially inform targeted interventions aimed at the subgroup of individuals most likely to benefit from trained immunity before a virus-specific vaccine. In the coming age of precision medicine, these stratified therapeutic strategies could have a powerful impact on disease control and prevention.

## Supplementary Material

ofaf007_Supplementary_Data

## Supplementary Data

Supplementary materials are available at *Open Forum Infectious Diseases* online. Consisting of data provided by the authors to benefit the reader, the posted materials are not copyedited and are the sole responsibility of the authors, so questions or comments should be addressed to the corresponding author.

## Notes

***Acknowledgments*.** We thank the entire BADAS clinical trial team at Texas A&M, Baylor College of Medicine, MD Anderson Cancer Center, and Cedars-Sinai Medical Center for their assistance in recruiting participants and completing this study, along with the participants, without whom this work could not have been completed.

***Financial support.*** This research was supported in part from funds provided by the Texas A&M University System and the National Institutes of Health (NIH) (grant numbers EB032983, AI170774, and AG078268). A. K., T. D. P., S. L. G., and C. C. were partially supported by the Cancer Prevention Institute of Texas (CPRIT) (grant numbers RP210227 and RP200504); the NIH (P30 shared resource grant CA125123); and the National Institute of Environmental Health Sciences (NIEHS) (grant numbers P30 ES030285 and P42 ES027725).
